# Supplementary material for: Influence of conspiracy theories and distrust of community health volunteers on adherence to COVID-19 guidelines and vaccine uptake in Kenya
Source: PLOS Glob Public Health. 2023 Mar 27;3(3):e0001146. doi: 10.1371/journal.pgph.0001146 (PMC10042357; doi:10.1371/journal.pgph.0001146)
Supplement: S1 Appendix — (DOCX) [file pgph.0001146.s001.docx]

S1 Appendix: The questionnaire and its frequency summary
