# Supplementary figures and images for: Influence of conspiracy theories and distrust of community health volunteers on adherence to COVID-19 guidelines and vaccine uptake in Kenya
Source: PLOS Glob Public Health. 2023 Mar 27;3(3):e0001146. doi: 10.1371/journal.pgph.0001146 (PMC10042357; doi:10.1371/journal.pgph.0001146)

S2 Appendix: COVID-19 conspiracy theories among non-self-confirmed CHVs


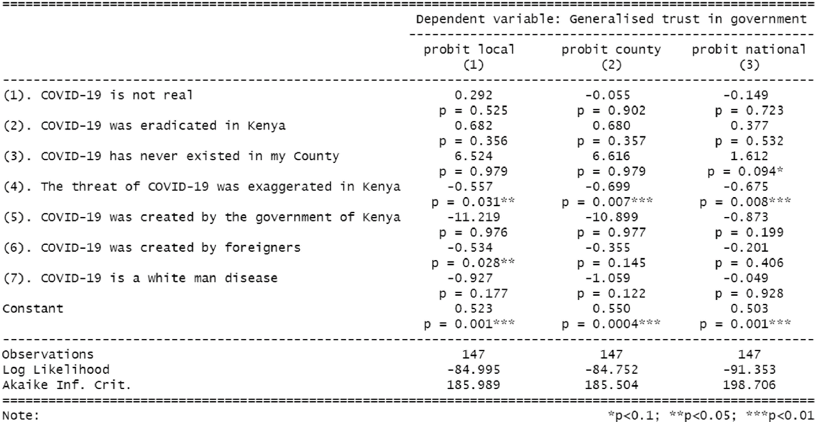

Supplement: S2 Appendix — (DOCX) [file pgph.0001146.s002.docx]

S3 Appendix: COVID-19-related trust among non-self-confirmed CHVs


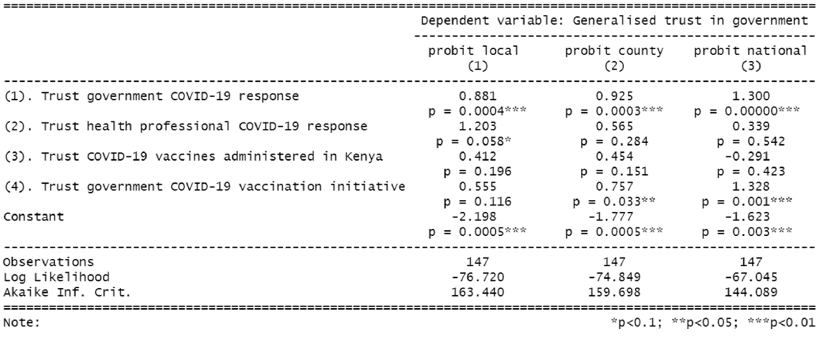

Supplement: S3 Appendix — (DOCX) [file pgph.0001146.s003.docx]

S4 Appendix: Perception of police enforcement of COVID-19 measures among non-self-confirmed CHVs


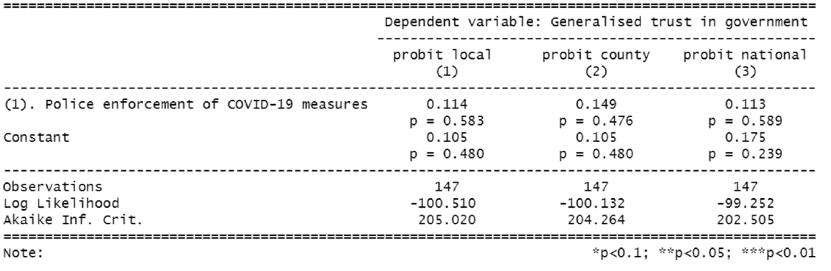

Supplement: S4 Appendix — (DOCX) [file pgph.0001146.s004.docx]

S5 Appendix: COVID-19 perceived risks among non-self-confirmed CHVs


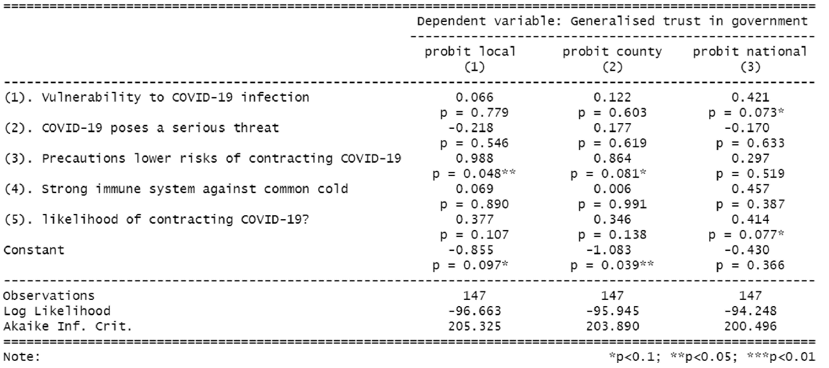

Supplement: S5 Appendix — (DOCX) [file pgph.0001146.s005.docx]
