# Supplementary material for: Influence of conspiracy theories and distrust of community health volunteers on adherence to COVID-19 guidelines and vaccine uptake in Kenya
Source: PLOS Glob Public Health. 2023 Mar 27;3(3):e0001146. doi: 10.1371/journal.pgph.0001146 (PMC10042357; doi:10.1371/journal.pgph.0001146)
Supplement: Attachment — Submitted filename: REBUTAL LETTER.docx [file pgph.0001146.s006.docx]

| Editor | Author |
| --- | --- |
| 1. Please amend your detailed Financial Disclosure statement. This is published with the article. It must therefore be completed in full sentences and contain the exact wording you wish to be published.  State the initials, alongside each funding source, of each author to receive each grant. | Done. Section 9. Funding on Page 19, last paragraph contains all the information regarding the grant. |
| 2. Please ensure that the funders and grant numbers match between the Financial Disclosure field and the Funding Information tab in your submission form. Note that the funders must be provided in the same order in both places as well. | Done. To be confirmed during the submission process |
| 3. Please update your Competing Interests statement. If you have no competing interests to declare, please state: “The authors have declared that no competing interests exist.” | Done. Section 8. Competing interests on page 19. |
| 4. In the online submission form, you indicated that “The data underlying this article will be shared upon reasonable request to the corresponding author.”. All PLOS journals now require all data underlying the findings described in their manuscript to be freely available to other researchers, either 1. In a public repository, 2. Within the manuscript itself, or 3. Uploaded as supplementary information. | Done. Access to data has been granted at  <https://doi.org/10.5281/zenodo.6554640> |
| 5. All main tables must be in an editable format for typesetting. Please include your tables in your main text as an editable table and not an image. | I am working on the editable tables |
| 6. Please provide separate figure files in .tif or .eps format and remove any figures embedded in your manuscript file. Please also ensure that all files are under our size limit of 10MB. | I will provide the tiff files |
| REVIEWER #1 |  |
| In the abstract, the rationale for this study should be strengthened along with what additional knowledge this paper is going to add to the existing literature. The timeline of the study is missing in the abstract. CHVs, should be defined before using them in the abstract. The abstract could be benefitted by discussing some quantitative data. | Done. See the Abstract on page 2 |
| The objective of the paper should clearly be mentioned in the last paragraph of the introduction. The literature review should be shortened and merged with the introduction. Follow the IMRAD. | Done. See the objective on page 7. |
| Who conducted the study is missing. The questionnaire, adapted from ---you should mention the study here not the number reference. | Done. Sub-section 3.4 Data collection method on page 8. |
| Hierarchical multiple logistic and probit regressions were done using (R script)3---You need to mention the version of the package. Rscript is not a software. | Done. See sub-section 3.5 Statistical methods on page 8 |
| In the result section, the data has been presented in way that makes it difficult to follow. | Advise on how we need to present the results. |
| The discussion should be started with a summary finding followed by supporting evidence from prior literature. | Not done |
| The subheadings in the discussion can be avoided. | Done. Sub-headings have been deleted in the discussion. |
| The tables can be provided in word. | To be converted from diagrams to editable word |
| You need to provide operational definitions of conspiracy theories and distrust. | Done. The operational definition for conspiracy theories is indicated in colour green on page 3, and trust in page 4. |
| REVIEWER #2 |  |
| 1. The author did not present any data in the abstract | Done. See the Abstract on page 2 |
| 2. The author included a section named “Literature review”. There is no such section recommended from this journal and not from any other journal that we know. This is a scientific manuscript NOT academic thesis. Please merge important information from literature review into Background section. | Done. The background and literature sections have been merged. The sub-sections previously on the literature review have been deleted. See pages3, 5 and 6 |
| 3. Methods: Please elaborate the statistical methods (3.5). Described how you have selected the variables for the model and how you have dropped them. I suggest you avoid sub-headings. | Done. The sub-headings under methods have been deleted. See Materials and methods.  The statistical methods undertaken were elaborated on pages 8 and 9 |
| 4. Please present the raw data i.e the value for n, N and % for each variable. This comment is applicable for tables 1-8 where applicable. The titles of most of the tables are vague – please be specific about the contents of the tables including duration, and locations of the study. In the current format of the table I find it very difficult to interpret the findings. What does the “Porbit local value” -0.259 for “COVID-19 in not real” means (Table 1, row 1)? The p-value 0.331. What does this mean? Does these mean the differences among the “Porbit local” participants? I am struggling to understand the values here. Please revise the table here and elsewhere in the manuscript. | The overall raw data is presented as Supplementary material 1  Am working on the tables. |
| 5. Discussion: please drop the sub-headings in the discussion section. | Done. Sub-headings have been deleted in the discussion. |
| 6. Conclusion: The author concluded that “Low income, family heads, job loss, pay cut, household members, urban residency, television news, gender, age, member of an association, education, religion, and marital status are potential covariates that influence generalised trust in government on matters COVID-19 restriction measures and vaccination.” It look very superficial conclusion. Let the readers know few important variables with their measure of strength that influences public trust. | To be summarised later |
