# Supplementary material for: Influence of conspiracy theories and distrust of community health volunteers on adherence to COVID-19 guidelines and vaccine uptake in Kenya
Source: PLOS Glob Public Health. 2023 Mar 27;3(3):e0001146. doi: 10.1371/journal.pgph.0001146 (PMC10042357; doi:10.1371/journal.pgph.0001146)
Supplement: Attachment — Submitted filename: REBUTAL LETTER.docx [file pgph.0001146.s007.docx]

| Editor | Author |
| --- | --- |
| 1. Please amend your detailed Financial Disclosure statement. This is published with the article. It must therefore be completed in full sentences and contain the exact wording you wish to be published.  State the initials, alongside each funding source, of each author to receive each grant. | Done. Section 8. FUNDING on Page 22 on the manuscript with tracked changes, contains all the information regarding the grant. |
| 2. Please ensure that the funders and grant numbers match between the Financial Disclosure field and the Funding Information tab in your submission form. Note that the funders must be provided in the same order in both places as well. | Done. The same information on page 22 of the manuscript with tracked changes, has been confirmed during the submission process under the financial disclosure field |
| 3. Please update your Competing Interests statement. If you have no competing interests to declare, please state: “The authors have declared that no competing interests exist.” | Done. Section 7. Competing interests on page 22 has been updated on the manuscript with tracked changes. |
| 4. In the online submission form, you indicated that “The data underlying this article will be shared upon reasonable request to the corresponding author.”. All PLOS journals now require all data underlying the findings described in their manuscript to be freely available to other researchers, either 1. In a public repository, 2. Within the manuscript itself, or 3. Uploaded as supplementary information. | Done. Access to data has been granted at  <https://doi.org/10.5281/zenodo.6554640>  and has been indicated on section 6 on page 21 of the manuscript with tracked changes. |
| 5. All main tables must be in an editable format for typesetting. Please include your tables in your main text as an editable table and not an image. | Done. All the 4 main editable table have been included in the main text. See pages 9, 10, 12 & 13 of the manuscript with tracked changes. |
| 6. Please provide separate figure files in .tif or .eps format and remove any figures embedded in your manuscript file. Please also ensure that all files are under our size limit of 10MB. | Done. All the figures embedded in the manuscript with tracked changes have been deleted. The conceptual framework, has been provided as a tiff file, and page 6 of the manuscript with tracked changes, has been highlighted where the figure will be inserted. |
| REVIEWER #1 |  |
| In the abstract, the rationale for this study should be strengthened along with what additional knowledge this paper is going to add to the existing literature. The timeline of the study is missing in the abstract. CHVs, should be defined before using them in the abstract. The abstract could be benefitted by discussing some quantitative data. | Done. The abstract has been revised accordingly. See the Abstract on page 2 of the manuscript with tracked changes. |
| The objective of the paper should clearly be mentioned in the last paragraph of the introduction. The literature review should be shortened and merged with the introduction. Follow the IMRAD. | Done. The literature review has been shortened and merged with the background. See pages 3-6 of the manuscript with tracked changes, including the inclusion of the objective on the last paragraph of section 1: Background |
| Who conducted the study is missing. The questionnaire, adapted from ---you should mention the study here not the number reference. | Done. See page 7, the first and the last paragraphs on section 2: Materials and methods on the manuscript with tracked changes |
| Hierarchical multiple logistic and probit regressions were done using (R script)3---You need to mention the version of the package. Rscript is not a software. | Done. See page 8 of the manuscript with tracked changes, the last paragraph of section 2: Material and Methods, line number 4 |
| In the result section, the data has been presented in way that makes it difficult to follow. | Done. The wordy contents on the previous results sections have been deleted and only the Probit model findings has been described. The findings for the Logit model have not been describe, but their tables have been provided as supplementary material 2-5. And the specific 4 main tables have been included in the main document to facilitate the easier flow of the contents. See pages 9, 10, 12 & 13 of the manuscript with tracked changes. |
| The discussion should be started with a summary finding followed by supporting evidence from prior literature. | Done. See page 14, the first paragraph of the manuscript with tracked changes |
| The subheadings in the discussion can be avoided. | Done. Sub-headings have been deleted in the discussion. See pages 14-21 of the manuscript with tracked changes. The discussion has also been beefed up with new content on socio demographics. |
| The tables can be provided in word. | Done. All the 4 main editable table have been included in the main text. See pages 9, 10, 12 & 13 of the manuscript with tracked changes. |
| You need to provide operational definitions of conspiracy theories and distrust. | Done. The operational definition for conspiracy theories is indicated on the first paragraph of page 4, while distrust is in page 4 paragraph 2. |
| REVIEWER #2 |  |
| 1. The author did not present any data in the abstract | Done. The abstract has been revised accordingly. See the Abstract on page 2 of the manuscript with tracked changes. |
| 2. The author included a section named “Literature review”. There is no such section recommended from this journal and not from any other journal that we know. This is a scientific manuscript NOT academic thesis. Please merge important information from literature review into Background section. | Done. The literature review has been shortened and merged with the background. See pages 3-6 of the manuscript with tracked changes, including the inclusion of the objective on the last paragraph of section 1: Background |
| 3. Methods: Please elaborate the statistical methods (3.5). Described how you have selected the variables for the model and how you have dropped them. I suggest you avoid sub-headings. | Done. The sub-headings under methods have been deleted. See Materials and methods on pages 6-8 of the manuscript with tracked changes.  The statistical methods undertaken were elaborated on pages 8 first paragraph of the manuscript with tracked changes, and section 3.2 Inferential statistics, the first paragraph on page 8 of the manuscript with tracked changes. |
| 4. Please present the raw data i.e the value for n, N and % for each variable. This comment is applicable for tables 1-8 where applicable. The titles of most of the tables are vague – please be specific about the contents of the tables including duration, and locations of the study. In the current format of the table I find it very difficult to interpret the findings. What does the “Porbit local value” -0.259 for “COVID-19 in not real” means (Table 1, row 1)? The p-value 0.331. What does this mean? Does these mean the differences among the “Probit local” participants? I am struggling to understand the values here. Please revise the table here and elsewhere in the manuscript. | Done. The overall raw data in the form of the value for n and the frequency for each variable has been presented as supplementary material 1.  The manuscript now contains 4 main tables showing the Probit model and their labels refined to give an easier meaning: local government; county government & national government. All the 4 main editable table have been included in the main text. See pages 9, 10, 12 & 13 of the manuscript with tracked changes. |
| 5. Discussion: please drop the sub-headings in the discussion section. | Done. Sub-headings have been deleted in the discussion. See pages 14-21 of the manuscript with tracked changes. The discussion has also been beefed up with new content on socio demographics. |
| 6. Conclusion: The author concluded that “Low income, family heads, job loss, pay cut, household members, urban residency, television news, gender, age, member of an association, education, religion, and marital status are potential covariates that influence generalised trust in government on matters COVID-19 restriction measures and vaccination.” It look very superficial conclusion. Let the readers know few important variables with their measure of strength that influences public trust. | The conclusion has been re-written again. See page 21. Equally, the new content to help the readers to understand better the role played by the significant socio demographics has been presented below Tables 2, 3, and 4 on pages 10, 12 & 13, respectively. Their descriptions has been interpreted in the discussion sections. See the 1st paragraphs on page 17 and 18, and pages 19 and 20. |
