# Supplementary material for: Influence of conspiracy theories and distrust of community health volunteers on adherence to COVID-19 guidelines and vaccine uptake in Kenya
Source: PLOS Glob Public Health. 2023 Mar 27;3(3):e0001146. doi: 10.1371/journal.pgph.0001146 (PMC10042357; doi:10.1371/journal.pgph.0001146)
Supplement: Attachment — Submitted filename: Rebutal Letter.docx [file pgph.0001146.s008.docx]

| Comments | Responses |
| --- | --- |
| Reviewer #2: Much better draft now. A few comments for further improvement. 1) Avoid using p values when you present 95% CI. You can keep the p-value in the table but don't need to show this in the abstract and any other text. | Done. See the tracked changes on pages 2, 15, 16, 17, 18, 19, 20 & 21 |
| 2) Raw data is still missing. You presented the Adjusted odds ratio which is fine but you also need to present the raw data (number and percentage in the table). For example in Table 2 .. "1) Trust government COVID-19 response" -- you presented few values [0.707, 1.071, 1.501] which need to clarify in table headings (is it odds ratio? If yes, please write in the table headings). The most important thing is that - you need to show how many people had trust on Government Covid-19 response and what percentage (n, and %). You can specify Big N for each variable separately or if same at the table legend/title. Then you can keep these odds ratio and p-value. People who don't have understanding on Odds ratio can still judge your findings based on the percentage. Please keep all these details in all four table (Table 1-4). | Done. Figure 2,3,4 & 5 shows the overall proportions. New tables with n and % have been added to the manuscript. See Table 1 (page 12); Table 2 (page 14); Table 4 (page 16); Table 6 (page 18) and Table 8 (page 19).  The sample size (n) appears on the title of the tables presenting the frequencies, while on the tables presenting the probit results, the sample size (n) has been added on the observations, together with a description of the adjusted odds ratios (AOR). See Table 3 (page 15); Table 5 (page 17); Table 7 (page 18-19) and Table 9 (page 21). |
| Reviewer #3: Abstract  The first two sentences in the abstract can be combined into one. | Done. The two sentences have been merged (See page 2). |
| The rationale is weak and needs to be strengthened. | Done. See Abstract page 2 |
| Who conducted the study should be mentioned explicitly. | Done. See Abstract page 2 |
| How were the respondents selected? | Done. See Abstract page 2 |
| The data analysis plan is missing in the abstract. | Done. See Abstract page 2 |
| Background The background needs to be shortened. The background section should discuss what knowledge the study will add to the existing literature. | Done. Background slightly reduced (See Pages 4, 5 & 6), but added content on knowledge and why it was important to study CHVs in Kenya (See page 7) paragraph 1. |
| Materials and methods The ethical aspects of the study could be discussed at the end of the methods section. | Done. See page 11, last paragraph |
| The background of the data collectors and their training should be discussed explicitly. | Done. See Page 10, paragraph 2 |
| The method section could be benefitted by following the chronological order • Study site and population.  • Outline the setting in which the study was carried out  • Mention the study participants. • Design and sampling • Describe the study design/approach  • Provide key operational definitions • Outline sampling methods • Data collection? • Outline data collection approaches | Done. The method section has been re-organised to fit the chronological order suggested. However, I have not included the sub-titles for the sections. Our first draft had sub-sections on the methods which the reviewers asked us to drop from the manuscript which we did. See reviewer comment on the Rebutal letter PGPH-D-22-00823_R1 |
| • Outline any special laboratory materials, equipment, or reagents. | Done. This section does not apply to our study. The study did not have any special laboratory materials, equipment, or reagents. |
| • Data analysis • Which variables were included in the multiple logistic regression, and why. | Done. See page 9 (paragraph 2) and page 11(paragraph 2) |
| Results: Socio-demographics need more data. Age, sex, education, and years as volunteers could be discussed here. | Done. A table on socio-demographics has been added on page 12 & 13 (See Table 1) and the description below it. |
| “The inferential statistics were evaluated and interpreted using the confidence level at 95% as a baseline”-could go under the method section. | Done. See page 10 last paragraph |
| 61.1% -could be spelled out. | Done. See page 3, section 3.2.1, first sentence |
| “conspiration theories”-Did you mean conspiracy theories? | Done. Yes, I meant conspiracy theories. Amended on page 15, paragraph 1, sentence 1 |
| “Household head (adjOR= 0.409, 95% CI: 0.213-0.785; p = 0.007) and gender (adjOR= 2.780, 95% CI: 1.526-5.064; p = 0.001), were significantly associated with trust in the COVID-19 vaccination initiative”. The idea is not clear here. We need to discuss the category. For example, Male gender was significantly associated ……………. | Done. Significant associations with socio-demographics have already been discussed based on the odds ratios of their sub-categories. The interpretations on the odds ratios have been simplified for easier comprehension by a layperson. See the parts under the discussion section marked in colour red on pages 24, 25, 26 and 27 on the manuscript with track changes. |
| Discussion: The authors need to use more references to support the statements discussed. “A good example was the allegation that the Nyanza region immigration officials had issued 25 foreigners from India to work at Kibos sugar and allied industries in Siaya county. Five of them tested positive for the delta variant of COVID-19” A reference is needed.  Page 18-foot noted could be deleted and used as a reference. | Done. All the foot noted references have been deleted and added to the references. See page 24 (Reference 39) and page 26 (References 40-49). |
| younger CHV—Age is needed here | Done. A descriptive Table 1 has been added to the proposal. It outlines the categories of the demographics used in the study (See Table 1 on page 12-13). Equally, a description of age has been added on page 24 (paragraph 1) and page 27 (paragraph 2). |
| The authors need to discuss the limitations of the study and focus on the impact of these limitations have on the conclusions. | Done. See limitations on page 28, last paragraph |
| Recommendations need to be strengthened. The authors could discuss the next steps that are practical and applicable to the context. What should specific research questions next be pursued? | Done. See recommendations on page 29, paragraph 2 |
